# Transarterial Embolization for Cystic Artery Pseudoaneurysm Caused by Hepatocellular Carcinoma Rupture in the Gallbladder: A Case Report

**DOI:** 10.7759/cureus.56400

**Published:** 2024-03-18

**Authors:** Soichiro Okamoto, Yusuke Matsui, Shoichi Komoto, Takao Hiraki

**Affiliations:** 1 Radiology, Tsuyama Chuo Hospital, Okayama, JPN; 2 Radiology, Faculty of Medicine, Dentistry, and Pharmaceutical Sciences, Okayama University, Okayama, JPN; 3 Internal Medicine, Tsuyama Chuo Hospital, Okayama, JPN

**Keywords:** hepatocellular carcinoma rupture, alcoholic liver cirrhosis, hemobillia, transarterial embolization, cystic artery pseudoaneurysm

## Abstract

We report the rare case of an 80-year-old man with hepatocellular carcinoma that ruptured in the gallbladder, causing a cystic artery pseudoaneurysm and hemobilia. Emergency transarterial embolization (TAE) successfully controlled the bleeding without causing ischemic cholecystitis. Cone-beam computed tomography angiography was useful in identifying the bleeding branch of the selectively embolized cystic artery. Although the patient had poor liver function (Child-Pugh class C) before TAE, it remarkably improved after embolization due to the resolution of coagulopathy and obstructive jaundice caused by hemobilia. TAE was considered useful for this rare clinical condition.

## Introduction

Spontaneous rupture of a hepatocellular carcinoma (HCC) is a potentially life-threatening condition with active arterial bleeding in the abdominal cavity in most cases. Although HCC causing hemobilia by rupturing the intrahepatic bile duct has been previously reported [1], HCC rupture in the gallbladder has rarely been reported. Here, we report a rare case of HCC rupture in the gallbladder, causing a cystic artery pseudoaneurysm that was successfully treated with transarterial embolization (TAE).

## Case presentation

An 80-year-old man presented at our hospital with epigastric pain and melena. The patient had a history of diabetes mellitus, hypertension, and heavy alcohol consumption. He had no significant family history or surgical history. At the time of presentation, the patient has a blood pressure of 153/61 mmHg, a heart rate of 100/min, and jaundice. Blood tests revealed the following abnormal values: hemoglobin of 7.8 g/dL, platelet count of 324 × 10^9^/L, total bilirubin of 9.5 mg/dL, direct bilirubin of 6.3 mg/dL, albumin of 2.5 g/dL, and prothrombin activity of 67.2%. The patient’s α-fetoprotein (AFP) was 2.4 ng/mL, and protein induced by vitamin K absence or antagonism factor II (PIVKA-II) was 22,625 mAU/mL (Table 1). The serological results for hepatitis B and C were negative.

**Table 1:** Laboratory results WBC, white blood cells; RBC, red blood cells; Hb, hemoglobin; Plt, platelet; AST, aspartate transaminase; ALT, alanine transaminase; ALP, alkaline phosphatase; T-bil, total bilirubin; D-bil, direct bilirubin; Alb, albumin; PT, prothrombin time; PIVKA-Ⅱ, protein induced by vitamin K absence or antagonism factor II; AFP, α-fetoprotein

Test	Patient's value	Reference value
WBC	11.8×10^3^/µL	3.3-8.6×10^3^/µL
RBC	1.92×10^6^/µL	4.35-5.55×10^6^/µL
Hb	7.8 g/dL	13.7-16.8 g/dL
Plt	324×10^3^/µL	158-348×10^3^/µL
AST	274 U/L	13-30 U/L
ALT	215 U/L	10-42 U/L
ALP	806 U/L	38-113 U/L
T-bil	9.5 mg/dL	0.4-1.5 mg/dL
D-bil	6.3 mg/dL	0-0.4 mg/dL
Alb	2.5 g/dL	4.1-5.1 g/dL
PT activity	67.2%	70-130%
PIVKA-Ⅱ	22625 mAU/mL	<40 mAU/mL
AFP	2.4 ng/mL	<10 ng/mL

**Figure 1:**
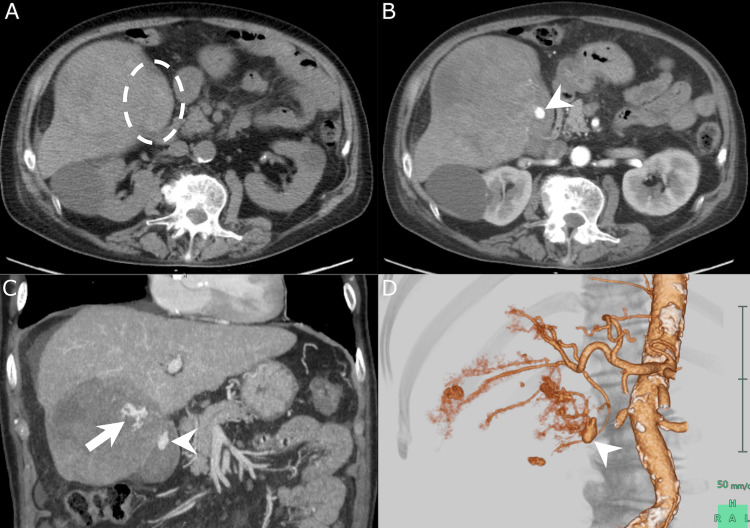
Non-contrast and contrast-enhanced CT image (A) A non-contrast-enhanced CT image demonstrates a hematoma in the gallbladder (white dotted circle). (B) An early phase contrast-enhanced CT image demonstrates a pseudoaneurysm in the gallbladder (white arrowhead). (C) A coronal maximum intensity projection image demonstrates extravasation of contrast media within a large hepatic tumor (white arrow) and a pseudoaneurysm in the gallbladder (white arrowhead). (D) A three-dimensional volume-rendered CT angiography image demonstrates a cystic artery pseudoaneurysm (white arrowhead). CT, computed tomography

Contrast-enhanced computed tomography (CT) revealed multiple hypervascular hepatic tumors consistent with HCC. Multiple intratumoral extravasations of contrast medium were found in the tumor, which was 12 cm in diameter and in contact with the gallbladder (Figure 1). A hematoma and pseudoaneurysm were also detected in the gallbladder (Figure 1).

**Figure 2:**
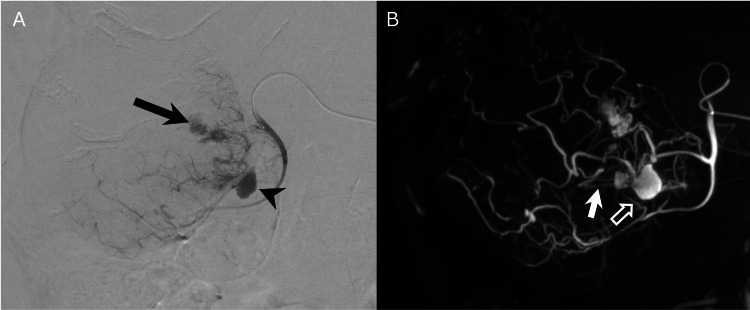
Angiogram and cone-beam computed tomography angiography from a cystic artery (A) An angiogram from a cystic artery demonstrates extravasation within the tumor (black arrow) and a pseudoaneurysm (black arrowhead). (B) Cone-beam computed tomography angiography demonstrates the branches from both the superficial (white hollow arrow) and deep (white arrow) cystic arteries, supplying the pseudoaneurysm.

The patient was diagnosed with ruptured HCC bleeding in the gallbladder with a cystic artery pseudoaneurysm. Although the patient was hemodynamically stable, we performed emergency embolization because his liver function might have been impaired due to bleeding in the biliary tract. Embolization of the cystic artery was considered feasible without affecting liver function. The ruptured tumor was mainly fed by the cystic artery. Angiography of the cystic artery demonstrated extravasation within the tumor and a cystic artery pseudoaneurysm (Figure 2A). The bleeding arterial branch was identified using cone-beam CT angiography of the cystic artery (Figure 2B).

**Figure 3:**
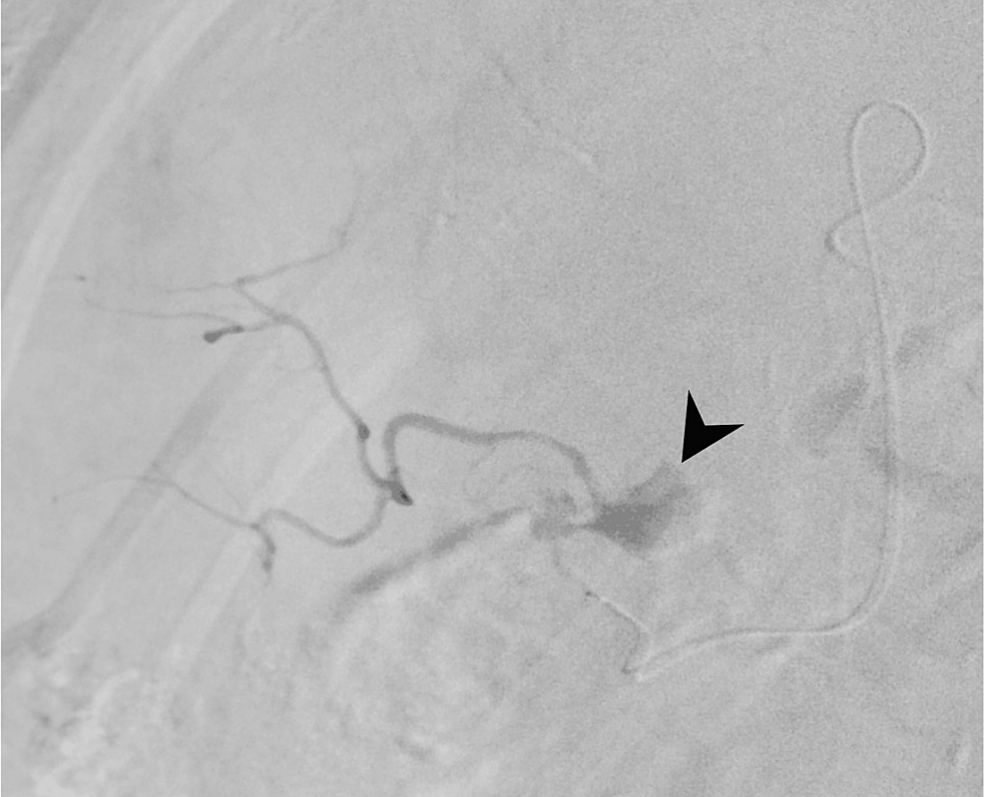
A selective angiogram of the branch of the superficial cystic artery A selective angiogram of the branch of the superficial cystic artery demonstrates a pseudoaneurysm in the gallbladder (black arrowhead).

After confirming the presence of the pseudoaneurysm by angiography of the superficial cystic artery branch (Figure 3), embolization of the branch was performed using 1.0 mL of a mixture of N-butyl cyanoacrylate (NBCA) and lipiodol in a 1:2 ratio.

**Figure 4:**
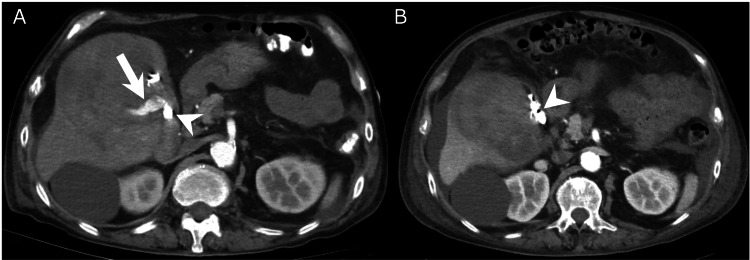
Contrast-enhanced CT image (A) An early phase contrast-enhanced CT image two days after the first transarterial embolization reveals residual extravasation in the gallbladder bed (white arrow). The n-butyl-2-cyanoacrylate cast in the pseudoaneurysm is also demonstrated (white arrowhead). (B) The follow-up contrast-enhanced CT 11 days after the second transarterial embolization demonstrates the n-butyl-2-cyanoacrylate cast in the gallbladder (white arrowhead) and no extravasation. There are no findings suggestive of ischemic cholecystitis. CT, computed tomography

Furthermore, the intratumoral extravasation from the deep cystic artery branch was selectively embolized using gelatin sponge particles. Two days later, follow-up CT revealed residual extravasation in the gallbladder bed (Figure 4A), which prompted a second TAE. Coil embolization of the superficial cystic artery branch was performed using three detachable coils, and NBCA embolization of the deep cystic artery branch was performed using 0.6 mL of a mixture of NBCA and lipiodol in a 1:3 ratio. Hemostasis was achieved. Follow-up CT showed the disappearance of the intratumoral extravasation and cystic artery pseudoaneurysm without ischemic cholecystitis (Figure 4B).

Liver function improved after TAE, reducing total bilirubin level from 9.5 mg/dL to 2.3 mg/dL within one week after the second TAE. Two weeks later, the patient developed an esophageal variceal rupture and underwent endoscopic variceal ligation. Follow-up blood tests at two months post-TAE showed remarkable improvements in relevant values, including hemoglobin of 12.1 g/dL, total bilirubin of 1.0 mg/dL, albumin of 3.2 g/dL, and prothrombin activity of 87.4%. The patient was discharged three months later and was alive six months post-TAE. Additional treatment, such as chemotherapy, for residual HCCs is currently being considered.

## Discussion

The gallbladder is normally located on the visceral surface of the liver and fits in the fossa between subsegments IV and V. Therefore, HCC in subsegments IV or V can potentially rupture in the gallbladder; however, this is rare. One possible reason for the rarity of HCC rupture in the gallbladder may be that HCC rarely invades the gallbladder because it barely destroys the muscular layer and collagen fibers of the gallbladder wall [2]. In the present case, the ruptured tumor was mainly fed by the cystic artery, and intratumoral extravasations from multiple branches of the cystic artery were detected. The small supplying arteries of the HCC have been reported to become stiff and brittle, facilitating rupture [3]. We speculated that the small cystic artery branch supplying the tumor ruptured between the gallbladder wall and the tumor in the gallbladder bed, resulting in bleeding in the gallbladder and pseudoaneurysm formation. The large size of the HCC and the patient’s hypertension may have also contributed to the rupture [4].

HCC patients with Child-Pugh class C disease were managed with supportive care according to a strategy based on the Barcelona Clinic Liver Cancer staging system [5]. However, emergency TAE has recently been reported to be effective in patients with ruptured HCC and Child-Pugh class C disease, providing better hemodynamic stabilization and a higher overall survival rate than conservative treatment [6]. In the present case of Child-Pugh class C disease, although the patient was hemodynamically stable, we performed emergency selective embolization of the cystic artery branches to treat the pseudoaneurysm in the gallbladder and intratumoral bleeding. Hemostasis was achieved, and liver function improved remarkably from Child-Pugh class C (11 points) to Child-Pugh class A (6 points) after embolization. Since only the cystic artery was embolized, there were no adverse effects on the liver function. We considered that the resolution of coagulopathy and obstructive jaundice caused by hemobilia, as well as the patient's abstinence from alcohol during hospitalization, contributed to the improvement in liver function.

Cystic arterial pseudoaneurysms secondary to acute cholecystitis or cholecystectomy have also been reported [7,8]. Cholecystectomy is recommended for the treatment of cystic arterial pseudoaneurysms caused by cholecystitis [8]. TAE for cystic artery pseudoaneurysms may be another option, particularly in patients with multiple comorbidities and high surgical risk. However, Kim et al. reported that ischemic cholecystitis was associated with a risk of mortality after TAE for cystic artery bleeding [9]. Therefore, careful observation is required after embolization of the cystic artery, and cholecystectomy should be considered if gallbladder ischemia is suspected.

In the present case, a cystic artery pseudoaneurysm was successfully embolized using TAE, without causing ischemic cholecystitis. Superselective embolization is recommended to avoid ischemic cholecystitis during TAE of HCC supplied by branches of the cystic artery branches [10]. There are multiple communications between the deep and superficial cystic arteries around the gallbladder body and fundus, and both arteries can supply the tumor [10]. In the present case, both branches of the superficial and deep cystic arteries were associated with a cystic artery pseudoaneurysm, and cone-beam CT angiography was useful for identifying the target arteries for superselective embolization.

## Conclusions

We report a case of cystic artery pseudoaneurysm caused by HCC rupture of the gallbladder. Superselective TAE of the branches of the cystic artery was successfully performed without causing ischemic cholecystitis. Liver function improved after TAE, presumably because of the resolution of coagulopathy and obstructive jaundice caused by hemobilia. TAE is useful for this rare clinical condition. This case report contributes to the understanding of this rare condition and highlights the potential of TAE as a minimally invasive procedure.

## References

[1] Ogura T, Okuda A, Higuchi K (2018). Hemobilia due to hepatocellular carcinoma: cholangioscopic findings and novel endoscopic hemostasis. Hepatobiliary Pancreat Dis Int.

[2] Ryu HS, Hwang ET, Choi CS, Kim TH, Kim HC, Yun KJ, Park DE (2009). [A case of hepatocellular carcinoma invading the gallbladder misdiagnosed as a primary gallbladder carcinoma] [Article in Korean]. Korean J Hepatol.

[3] Zhu LX, Geng XP, Fan ST (2001). Spontaneous rupture of hepatocellular carcinoma and vascular injury. Arch Surg.

[4] Sahu SK, Chawla YK, Dhiman RK (2019). Rupture of hepatocellular carcinoma: a review of literature. J Clin Exp Hepatol.

[5] Forner A, Reig ME, de Lope CR, Bruix J (2010). Current strategy for staging and treatment: the BCLC update and future prospects. Semin Liver Dis.

[6] Fan WZ, Zhang YQ, Yao W (2018). Is emergency transcatheter hepatic arterial embolization suitable for spontaneously ruptured hepatocellular carcinoma in Child-Pugh C cirrhosis?. J Vasc Interv Radiol.

[7] Taghavi SM, Jaya Kumar M, Damodaran Prabha R, Puhalla H, Sommerville C (2021). Cystic artery pseudoaneurysm: current review of aetiology, presentation, and management. Surg Res Pract.

[8] Patil NS, Kumar AH, Pamecha V, Gattu T, Falari S, Sinha PK, Mohapatra N (2022). Cystic artery pseudoaneurysm-a rare complication of acute cholecystitis: review of literature. Surg Endosc.

[9] Kim HC, Jeong YS, Han K, Kim GM (2023). Transcatheter arterial embolization of cystic artery bleeding. Front Surg.

[10] Kim HC, Miyayama S, Choi JW, Kim GM, Chung JW (2023). Hepatocellular carcinoma supplied by the inferior phrenic artery or cystic artery: anatomic and technical considerations. Radiographics.

